# Sulfur-Doped Alkylated Graphene Oxide as High-Performance Lubricant Additive

**DOI:** 10.1186/s11671-020-3257-7

**Published:** 2020-01-30

**Authors:** Jun Ma, Yunpeng Xiao, Yuanbao Sun, Jianqiang Hu, Yuelun Wang

**Affiliations:** 1 Air Force Logistics College, Xuzhou, 221000 People’s Republic of China; 2 Key Laboratory of Coal Processing and Efficient Utilization, Ministry of Education, China University of Mining & Technology, Xuzhou, 221116 People’s Republic of China

**Keywords:** Sulfur-doped alkylated graphene oxide, Chemical modification, Dispersity, Anti-wear additive, Lubrication oil

## Abstract

Sulfur-doped graphene oxide (SA-GO) prepared by sulfuration and alkylation of graphene oxide is applied as an efficient green anti-wear additive for harsh operation conditions of engines. X-ray photoelectron spectroscopy analysis reveals the sulfur content of octadecylamine-modified SA-GO (sulfuration follows alkylation) is increased by 79 times compared with the reverse process that alkylation follows sulfuration, suggesting the preparation route is a key factor of the sulfuration process. The higher sulfur content and –C–S–C– sulfur bonding constitution result better lubrication effect, while the investigation of chain length of alkylation modification and concentration of the alkylated sulfur-doped graphene oxide indicates the octylamine-modified SA-GO shows smaller diameter of wear scar within the concentration range between 1 × 10^−4^ and 2.5 × 10^−4^ wt%. The decrement percent of wear scar diameter is 43.2% in 928 lubrication oil and 17.2% in PAO4 oil while the SA-GO modified by octylamine is applied with the concentrations 2.5 × 10^−4^ wt% in PAO4 and 1 × 10^−4^ wt% in 928 oil, respectively. The sulfur content in oil samples is only 0.006~0.001 wt%, which is much lower than the sulfur content standard recommended by ILSAC that is 0.5 wt%. The research work indicates the SA-GO additive is more feasible for the pollution treatment which focuses the substantial reduction of sulfur content in lubrication oil on the premise of improving lubrication capability.

## Introduction

Sulfur-based organic anti-wear additives are widely used in the application of lubrication oils to promote the anti-wear ability of friction pairs under extreme pressure in which oil membrane failed to separate the moving parts, such as sulfur-containing gear oil [1]and poly-α-olefin [2]. Since the excessive active sulfur in organic compounds could poison the ternary catalyst of emission reduction system while serving on the piston ring and cylinder wall, which has been resulting to serious environment pollution, the coming mandatory standard from the International Lubricant Standardization and Approval Committee (ILSAC) [3] requires the sulfur content of lubrication oil should be less than 0.5 wt%, for the reason that the higher sulfur content in lubricant could deteriorate the quality of engine exhaust gas [4]. In order to resolve the problem, many strategies including the organic friction modifier such as sulfur-free alkyl-cyclens [5], quinolinium salts [6], and nanomaterial anti-wear additives like BN co-doped graphene [7], SiC@graphene [8], crumpled graphene [9], and graphene nanoscroll [10] have been developing to reduce or remove the sulfur content of lubrication oils. However, the above developing methods still need a long time to verify the practical lubrication effect, environment factor, safety, and other aspects to finally confirm the actual application results.

This paper focuses to prepare the sulfur-doped graphene oxide to act as an efficient low-sulfur content anti-wear additive for harsh operation conditions at high temperature. Based on the preparation researches of sulfur-doped graphene, the unique nanomaterials could be obtained by the reactions of thiourea [11], NaHSO_3_ [12], K_2_S_2_O_8_ [13], phenyl disulfide [14, 15], sulfur [16], Na_2_S [17], CS_2_ [18], P_4_S_10_ [19], H_2_S [20], SO_2_ [21], CS_2_ [21], benzyl disulfide [22], and graphene oxide under the hydrothermal or other simple conditions. After the sulfur doping process, sulfur-doped graphene oxide could significantly improve the anti-wear properties of the based oil. The most important feature of the unique anti-wear additive is the sulfur element that was anchored in the molecular structure of sulfur-doped graphene oxide. Adding the sulfur-doped graphene oxide in lubrication oil would simultaneously keep the advantages of sulfur-containing anti-wear additives and reduce the sulfur content to ~ 1/1000 compared with organic sulfur additives.

## Results and Discussion

**Fig. 1:**
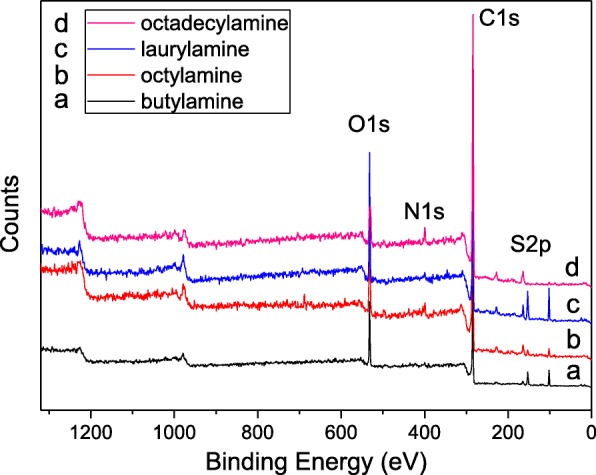
The XPS survey of the SA-GO which is prepared by the reactions of butylamine (**a**), octylamine (**b**), laurylamine (**c**), octadecylamine (**d**), and the sulfur-doped graphene oxide (the oxidation time of the graphene oxide is 24 h)

The XPS analysis (Fig. 1) suggests that the four elements, oxygen, nitrogen, carbon, and sulfur, exist in the sulfur-doped graphene oxide. Two different preparation routes applied in the research indicate that the modification method would substantially affect the chemical composition of the sulfur-doped alkylated graphene oxide.

The SA-GO and AS-GO were examined by XPS to evaluate the sulfur doping efficiency of the preparation routes. In comparison with the sulfur content of AS-GO, the sulfur doping process of SA-GO is much better than that of AS-GO. As shown in Table 1, the sulfur content of SA-GO (1.94–3.16 at%) is a few dozen times than that of AS-GO (0.04–0.08 at%). The results also indicate that the alkylation before sulfuration would dramatically reduce the active points on the graphene oxide, which results significant efficiency decrement of the followed sulfuration of AS-GO. Since the sulfur content of AS-GO is within the range of 0.04–0.08 at%, the preparation of the AS-GO shows rare advantage and alkylamine selectivity of the sulfur doping process efficiency. As shown in Table 1, the sulfur content of octadecylamine-modified SA-GO is increased by 79 times versus octadecylamine-modified AS-GO.

**Table 1:** The chemical composition of SA-GO and AS-GO determined by XPS (at%)

Species	Elements	Butylamine	Octylamine	Laurylamine	Octadecylamine
SA-GO	C	79.74	82.17	80.27	82.41
	O	14.49	11.71	13.51	10.88
	N	3.83	3.63	3.42	3.55
	S	1.94	2.49	2.8	3.16
AS-GO	C	92.15	89.73	89.93	89.84
	O	7.41	9.79	9.61	9.47
	N	0.36	0.42	0.42	0.65
	S	0.08	0.06	0.04	0.04

Nitrogen content is also affected by the preparation routes of sulfur-doped graphene oxide. Being firstly modified by alkylamine and then with P_4_S_10_, the atomic percent of nitrogen in AS-GO is just 0.36–0.65 at%, which is obviously lower than that of SA-GO (3.42–3.83 at%). However, the nitrogen in SA-GO and AS-GO is much different from the nitrogen of the nitrogen doping graphene. The nitrogen of SA-GO and AS-GO is mainly located at the functional groups of alkylamine, not in the structure of graphene. However, based on the SA-GO added amount of 1~5 × 10^−4^ wt% in oil samples, the sulfur content in oil samples is only 0.006~0.001 wt%, which is much less than the sulfur content standard of 0.5 wt% from ILSAC [3].

**Fig. 2:**
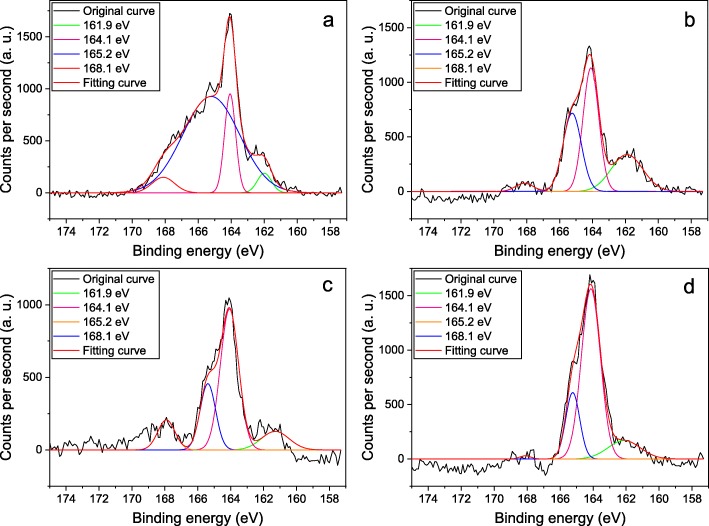
The high-resolution sulfur (2p) XPS analysis of the SA-GO which is prepared by the reactions of butylamine (**a**), octylamine (**b**), laurylamine (**c**), octadecylamine (**d**), and the sulfur-doped graphene oxide (the oxidation time of the graphene oxide is 24 h)

The peak fitting results show that the bond content is much different in the SA-GO after sulfur-doped graphene oxide reacted with butylamine, octylamine, laurylamine, and octadecylamine. In the high-resolution S2p analysis of SA-GO (Fig. 2), two peaks centered at 161.9 eV and 164.1 eV should be assigned to S2p_3/2_ and S2p_1/2_, respectively, which are the peaks resulted from the S2p spin-orbit doublet of the –C–S–C– bond [11, 13]. The S2p_3/2_ and S2p_1/2_ bonding configurations can be attributed to the formation of C=S and C–S bonds in the structure of the SA-GO [16]. The other two peaks at around 165.2 eV and 168.1 eV can be assigned to –C–SO_x_–C– bonding, which is mainly derived from sulfur oxide species in SA-GO [15–17, 22]. Based on the peak fitting results from Fig. 2, the –C–S–C– and –C–SO_x_–C– bonding configurations in the SA-GO are calculated and demonstrated in Table 2. After the sulfuration and alkylation of the GO (the GO is prepared by the oxidation for 24 h), the atomic percent of –C–S–C– bond is quite similar in the SA-GO that octylamine, laurylamine, and octadecylamine are used as reagent for alkylation.

**Table 2:** The bonding configurations in the SA-GO (at%)

Bond type	Amine species			
	Butylamine	Octylamine	Laurylamine	Octadecylamine
–C–S–C–	19.07	66.26	66.77	61.67
–C–SO_x_–C–	80.93	33.74	33.23	38.33

The SA-GO is prepared by the reactions of butylamine, octylamine, laurylamine, octadecylamine, and the sulfur-doped graphene oxide (the oxidation time of the graphene oxide is 24 h)

Although the sulfur content significantly increases after sulfuration, the –C–S–C– bond configuration indicates the bonding of sulfur in the molecular structure of graphene oxide, and the –C–SO_x_–C– bond configuration is attributed to the incomplete reduction reaction in the sulfuration while P_4_S_10_ applied to react with graphene oxide. In the high-resolution sulfur XPS analysis, the butylamine modified graphene oxide shows the lowest content of –C–S–C– among the four alkylamines applied in this paper. The results indicated that the subsequent alkylation process could affect the C–S bonding configurations.

**Fig. 3:**
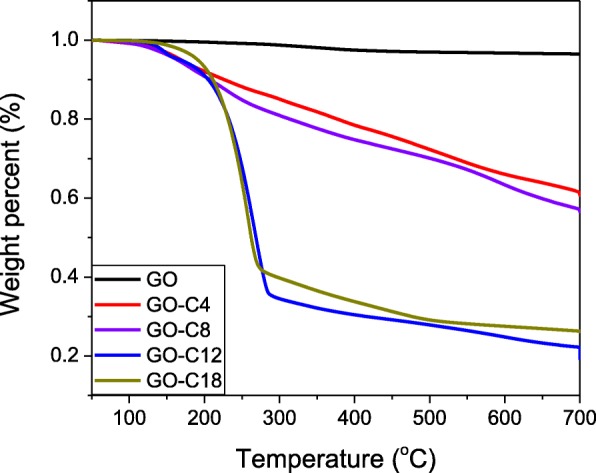
The TGA analysis of SA-GO which is prepared by the reactions of butylamine (GO-C4), octylamine (GO-C8), laurylamine (GO-C12), octadecylamine (GO-C18), and the sulfur-doped graphene oxide (the oxidation time of the graphene oxide is 24 h)

The thermogravimetric analysis (TGA) is applied to determine the alkylation efficiency of the reactions between the butylamine (GO-C4), octylamine (GO-C8), laurylamine (GO-C12), octadecylamine (GO-C18), and the sulfur-doped graphene oxide (the oxidation time of the graphene oxide is 24 h). As shown in Fig. 3, the weight loss of GO-C12 (80.9 wt%) and GO-C18 (73.9 wt%) is much higher than that of GO-C4 (39.3 wt%) and GO-C8 (42.6 wt%) and indicates that the content of the chemically grafted organic compounds of GO-C12 and GO-C18 is much higher. However, the grafted organic compounds of GO-C4 and GO-C8 are even high, since the weight loss of GO is only 3.5 wt% at 700 °C which suggests nearly no organic compounds exist in GO.

**Fig. 4:**
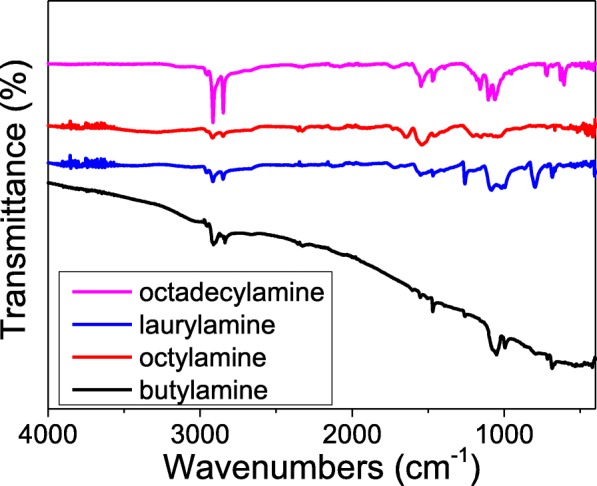
The ATR-FTIR spectra of the SA-GO which is prepared by the reactions of butylamine, octylamine, laurylamine, octadecylamine, and the sulfur-doped graphene oxide (the oxidation time of the graphene oxide is 24 h)

The alkylating of SA-GO also could be confirmed by ATR-FTIR spectra shown in Fig. 4. Strong absorption peaks located at ~ 2848 cm^−1^ and ~ 2780 cm^−1^ are assigned to the stretching vibration of C–H bonds of –CH_3_ and –CH_2_ groups, which is coincident with the results of TGA that the alkylated sulfur-doped graphene oxide contains considerable amount of organic matter. The peak centered at ~ 1540 cm^−1^ represents the out-plane vibration of the –CH_2_ group and asymmetric deformation vibration. And the broad and intensive absorption peak at ~ 1050 cm^−1^ is attributed to the stretching vibration of the –C–N bond, which composes the amido linkage (CO–NH) between the graphene oxide and alkylamines. The results of ATR-FTIR (Fig. 4) show that the alkylation process is effective for the preparation of alkylated sulfur-doped graphene oxide.

**Fig. 5:**
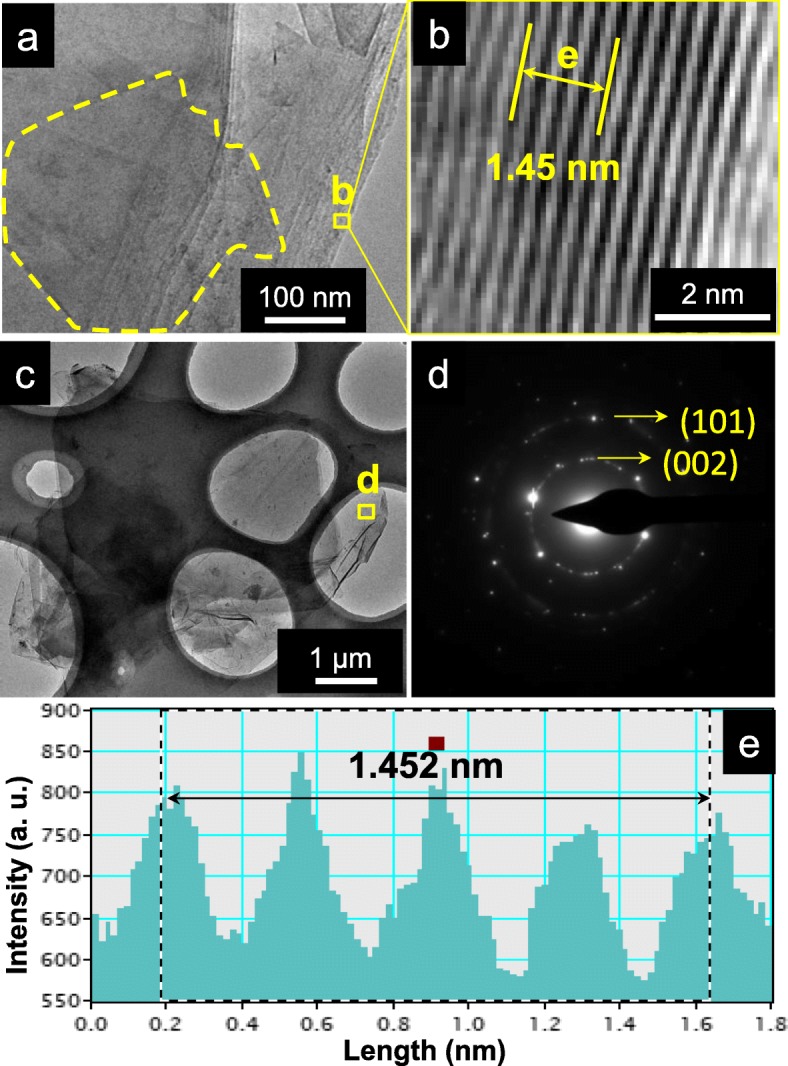
The TEM analysis of SA-GO (which is prepared by the reactions of octadecylamine and the sulfur-doped graphene oxide; the oxidation time of the graphene oxide is 24 h). Including the stacked SA-GO (**a**), the high-resolution TEM image of the square zone marked in **a** (**b**), the dispersed SA-GO (**c**), the SAED diffraction pattern of the square zone marked in **c** (**d**), and the crystalline space analysis of the position marked in **b** (e)

The TEM images and profile analysis of the typical SA-GO nanostructure are shown in Fig. 5. After filtration, the SA-GO is stacked in Fig. 5a; however, a graphene sheet-like nanostructure still could be delineated by a dash line zone. Figure 5b is the high-resolution TEM image of the square zone highlighted in Fig. 5a. According to the profile analysis (Fig. 5e), the five layers measured in Fig. 5b is 1.452 nm, and thus the average layer distance is 0.363 nm, which is highly coincident to the interplanar spacing of graphite (JCPDS card no. 75-1621). Selected area electron diffraction (SAED) patterns (Fig. 5d) of SA-GO are nearly diffraction rings of graphite [23]. According to the JCPDS card no. 75-1621, the inner diffraction ring should be attributed to the (002) crystalline plane and the outer one is assigned to the (101) plane, which suggests the graphene nature of the SA-GO.

**Fig. 6:**
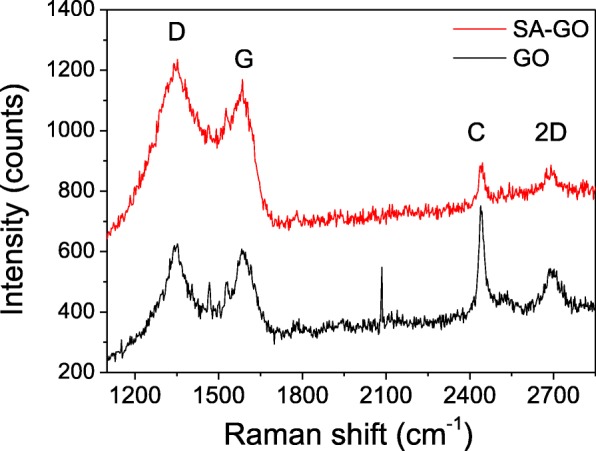
The typical Raman spectrum of SA-GO (which is prepared by the reactions of octadecylamine and the sulfur-doped graphene oxide; the oxidation time of the graphene oxide is 24 h) and GO (the oxidation time of the graphene oxide is 24 h)

According to the Raman analysis of the SA-GO (Fig. 6) which is prepared by the reactions of octadecylamine and the sulfur-doped graphene oxide (the oxidation time of the graphene oxide is 24 h), the two peaks centered at 1350 and 1584 cm^−1^ could be attributed to the D and G band of SA-GO, while the peak around 2690 cm^−1^ is assigned to the 2D bands of the SA-GO [23, 24]. The Raman peak at around 2440 cm^−1^ is suggested as the C [25] or D + D″ [26] band of graphene and could be seen in the Raman spectra in papers [27, 28]. The I_D_/I_G_ value of GO is 0.986, which is slightly lower than that of SA-GO (I_D_/I_G_ = 1.05), and indicates that the graphene structure in the modification reactions have not significantly changed.

**Fig. 7:**
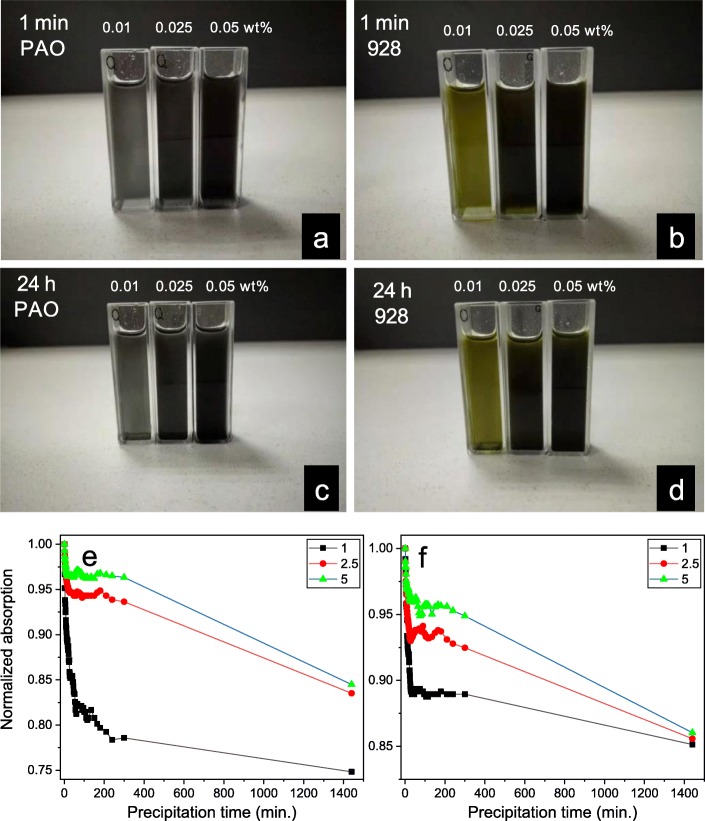
The optical photographs of the SA-GO (which is prepared by the reactions of octadecylamine and the sulfur-doped graphene oxide; the oxidation time of the graphene oxide is 24 h) ultrasonically dispersed in the PAO4 base oil (**a**) and the 928 aviation lubrication oil (**b**) and the PAO4 (**c**) and 928 (**d**) oil sample stand still for 24 h, respectively; the normalized adsorption of the SA-GO in PAO4 (**e**) and 928 (**f**) oil samples (**a**–**d**, examined by UV-vis spectrophotometer) with the concentration of SA-GO is 1, 2.5, and 5 × 10^−4^ wt% within 1440 min

The base oil of 928 aviation lubrication oil is mainly poly-α-olefin, PAO, which are saturated alkanes that have ~ 30 carbon atoms. In this paper, the 928 aviation lubrication oil and PAO4 (the kinematic viscosity is ~ 4 mm^2^/s at 100 °C) are used as substrate, and then the alkylated sulfur-doped graphene oxide is added into the oils, respectively, to explore their dispersing properties. As shown in Fig. 7, the photos of SA-GO oil samples showed that the color of oil samples gradually became darker with the increasing amount of SA-GO. This should be the increasing absorption of visible light since the SA-GO was added into the oils. However, there is a little sediment on the bottom of the cuvettes even if the graphene oxide has been chemically modified to improve the dispersity. As shown in Fig. 7b and d, the oil samples’ color is light yellow compared with the PAO4 oil samples’ color that is because the color of the 928 lubrication oil is light yellow. Apparently, the concentration of the SA-GO could substantially affect the absorption intensity.

In order to quantitatively analyze the dispersity of the SA-GO oil samples, the UV-vis spectrophotometer is applied to test the absorption of the SA-GO oil samples. The results are shown in Fig. 7e and f. After precipitation for 24 h (1440 min), the normalized absorption of the SA-GO oil samples is decreased. Interestingly, the reduction of normalized absorption of high concentration oil sample is relatively slower than that of the low concentration oil samples. The phenomenon indicates the substantially improved dispersity of the SA-GO which did not appear obvious aggregation even under the condition of a relatively high concentration.

**Fig. 8:**
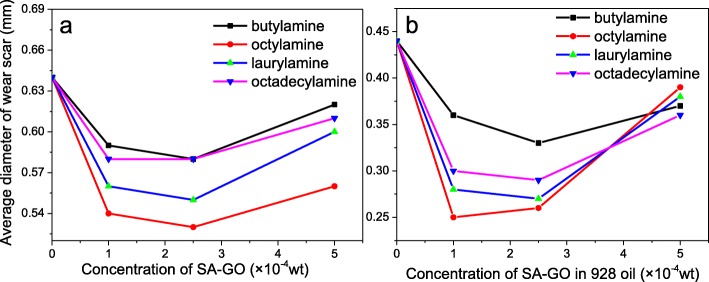
The average diameter of wear scar while the SA-GO (prepared by sulfur-doped GO and butylamine, octylamine, laurylamine, and octadecylamine; the oxidation time of the GO is 24 h) is applied as lubrication additive in PAO4 (**a**) and 928 (**b**) oils

From the decreasing tendency of the curve in Fig. 7e and f, a method of calculating the slope of the linear fitting curve of the linear tail end of the normalized adsorption of the SA-GO could quantitatively demonstrate the dispersity. As shown in Table 3, the calculation results indicate that the decreasing tendency of high concentration SA-GO oil samples is higher than low concentration SA-GO oil samples, even though the reduction of normalized absorption of high concentration oil sample is relatively slower than that of the low concentration oil samples. The dispersion analysis suggests that the SA-GO has excellent dispersity after chemical modification even at a relatively high concentration; however, the high concentration SA-GO oil samples have higher decreasing tendency in a long term. The tribological properties of SA-GO oil samples are measured by four-ball testers for evaluating the lubrication enhancement effect of SA-GO which was performed as a lubricant additive. As mentioned in the “Method” section, the diameter of wear scars is measured after testing is finished, and the maximum nonseizure load (*P*_*B*_) value is also acquired. The average diameter of the wear scars while the SA-GO is applied as lubrication additive is shown in Fig. 8.

**Table 3:** The slope of the linear fitting curve of the linear tail end of the normalized adsorption of the SA-GO (prepared by sulfur-doped GO and octadecylamine, the oxidation time of the GO is 24 h) in PAO4 and 928 oils (× 10^−5^)

Concentration	PAO4	928
0.01	− 3.1	− 3.2
0.025	− 9.1	− 6.3
0.05	− 10.0	− 8.6

Firstly, the average diameter of SA-GO 928 oil samples is much smaller than that of SA-GO disperse in PAO4 base oil. This phenomenon could be an attribute to the 928 lubrication oil that contains organic phosphate anti-wear additives which could effectively reduce the wear volume during sliding [29]. The phosphate-containing anti-wear additive could react with the steel friction pairs to generate tribofilm including iron phosphate, ferrous phosphate, and other phosphate-containing compounds under a boundary lubrication regime.

Secondly, the modification route plays an important role in the anti-wear behavior of SA-GO oil samples [30, 31]. The octylamine-modified SA-GO shows better lubrication properties among the oil samples including both PAO4 and 928 lubrication oils. The results are coincident with the analysis of TGA shown in Fig. 4 and the molecular structure of PAO4 or the base oil of the 928 lubrication oil. According to the TGA, the weight loss of SA-GO (modified by octylamine) is only about half compared with the SA-GO modified by laurylamine and octadecylamine, which means the amount of sulfur-doped graphene in the laurylamine- and octadecylamine-modified SA-GO is only about half compared with SA-GO modified by octylamine. Since the added amount of SA-GO is just 1, 2.5, and 5 × 10^−4^ wt%, respectively, the serious shortage of sulfur-doped graphene in the laurylamine- and octadecylamine-modified SA-GO will result in the deterioration of their anti-wear ability. On the other hand, the butylamine-modified SA-GO has similar sulfur-doped graphene content with the SA-GO modified by octylamine according to the TGA analysis. However, the dispersity of butylamine-modified SA-GO is intrinsically lower than octylamine-modified SA-GO since the alkylated carbon chain of octylamine-modified SA-GO is quite close to the side chain of PAO4 or the base oil of the 928 lubrication oil [32].

Thirdly, the concentration of SA-GO in the oil samples could affect the anti-wear capacity. Many researches have proved that if the concentration of graphene and/or its derivatives is too high, the graphene (or the derivatives) usually tends to aggregate in liquid. In lubrication application, the aggregated graphene could not be performed as an effective lubrication additive, even harmful towards the tribological properties. In this case, the SA-GO concentration of 5 × 10^−4^ wt% is too high for lubricating application both in 928 and PAO4 oils, due to the abnormal increase of wear scar diameter. Thus, the SA-GO (modified by octylamine) concentrations of 1 and 2.5 × 10^−4^ wt% are confirmed in the research that have better lubrication effect in 928 lubrication oil (the wear scar diameter is 0.25 mm) and PAO4 oil (the wear scar diameter is 0.53 mm), respectively. Compared with the pure 928 lubrication oil and PAO4 oil, the decrement of wear scar diameter is 43.2% and 17.2% in the 928 lubrication oil and PAO4 oil, respectively. The lubrication enhancement effect of SA-GO in the 928 lubrication oil is much better than that in PAO4 base oil, which demonstrates the excellent synergistic lubrication effect of SA-GO in the 928 lubrication oil.

**Fig. 9:**
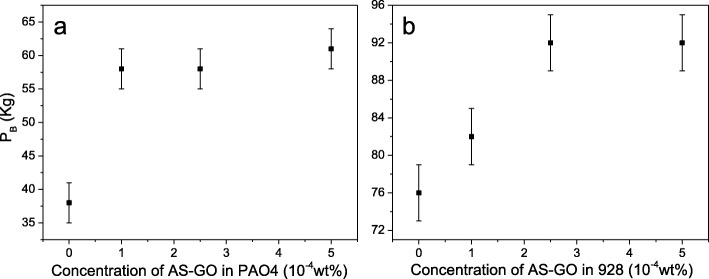
**a**, **b** The maximum nonseizure load (PB) value while SA-GO (prepared by sulfur-doped GO and octylamine, the oxidation time of the GO is 24 h) is applied as lubrication additive with certain concentration in PAO4 (**a**) and 928 (**b**) oil

The maximum nonseizure load (*P*_*B*_) value of the SA-GO (prepared by sulfur-doped GO and octylamine) in PAO4 (Fig. 9a) and 928 (Fig. 9b) lubrication oils was determined by the four-ball tester. As shown in Fig. 9a, the *P*_*B*_ enhancement of the SA-GO is quite considerable which has an increment of 55.6% for the *P*_*B*_ value at the concentration of 1 × 10^−4^ wt% and 72.2% for *P*_*B*_ value at the concentration of 5 × 10^−4^ wt%. The results of *P*_*B*_ value suggest that the oil membrane strength is substantially enhanced since the SA-GO was added into the PAO4 base oil samples. The *P*_*B*_ values of the SA-GO prepared by sulfur-doped GO, butylamine (Additional file 1: Figure S2a), laurylamine (Additional file 1: Figure S2b), and octadecylamine (Additional file 1: Figure S2c) in PAO4 are shown in SI, which indicate the similar results that the higher concentration of SA-GO in PAO4 would be helpful for the promotion of the maximum nonseizure load value.

The *P*_*B*_ value of the SA-GO (prepared by sulfur-doped GO and octylamine) in 928 lubrication oils (Fig. 9b) also increases with an increasing concentration of the SA-GO until 2.5 × 10^−4^ wt%. Even more, SA-GO is added into the 928 lubrication oil, but the *P*_*B*_ value of the SA-GO oil sample was unchanged at the concentration of 5 × 10^−4^ wt%. Compared with the SA-GO prepared by sulfur-doped GO and butylamine (Additional file 1: Figure S3a), laurylamine (Additional file 1: Figure S3b), and octadecylamine (Additional file 1: Figure S3c) in the 928 lubrication oil, the butylamine-modified SA-GO has relatively lower *P*_*B*_ value (82 kg) at the concentration of 2.5 × 10^− 4^ wt%. However, the laurylamine- and octadecylamine-modified SA-GO in 928 lubrication oil show similar *P*_*B*_ value (92 kg) with the octylamine-modified SA-GO. The phenomenon suggests that the added amount of SA-GO should be 2.5 × 10^−4^ wt% for considering the aggregation effect, anti-wear properties, and *P*_*B*_ value.

**Fig. 10:**
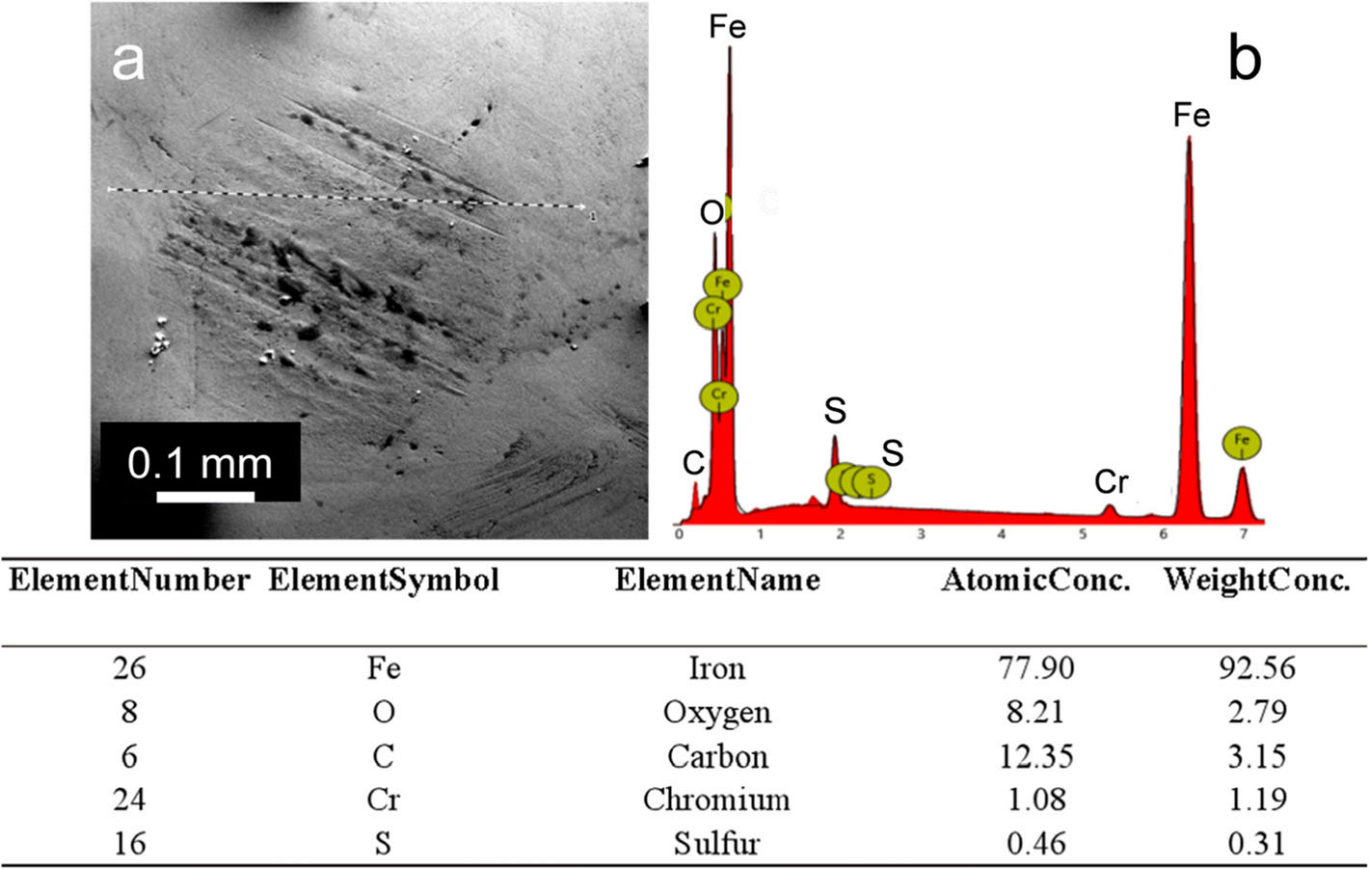
The SEM image (**a**) and EDS elemental analysis (**b**, the integration results of the line scanning shown in **a** which is marked as a dash line) of the wear scar while the SA-GO (prepared by sulfur-doped GO and octylamine; the oxidation time of the GO is 24 h and the concentration of SA-GO is 0.025 wt%) acted as lubricant additive in the 928 lubrication oil

After friction sliding, the SEM and EDS analysis of wear scar indicates the sulfur from SA-GO probably plays a key role in the reactive lubrication behavior to form sulfur-containing chemical boundary lubricating film. As shown in Fig. 10a (SEM image of wear scar) and b (EDS spectrum of Fig. 10a, the integration results of the line scanning shown in Fig. 10a which is marked as a dash line), the EDS spectrum of wear scar depicts five elements including Fe, O, C, Cr, and S that exist in the wear scar.

The sulfur content of wear scar reaches as high as 0.46 at% which is much higher than that of the wear scar from the pure 928 applied as lubricant (the sulfur content is at ~ 0%, Additional file 1: Figure S4). Thus, the higher sulfur content in tribofilm is strongly related to the average diameter of wear scar analysis, the dispersion study, and the TGA analysis, which suggest the anti-wear properties favor the higher sulfur content and higher graphene weight percent, and the dispersity benefits from the carbon chain length similar to the side chain of base oils.

## Conclusions

The SA-GO is prepared by the sulfuration and alkylation of graphene oxide. Based on the XPS analysis, the sulfuration follows the alkylation route (the product is SA-GO) that is much better than alkylation than follows sulfuration (the product is AS-GO) for the sulfur doping of graphene oxide. Anti-wear testing reveals that the SA-GO prepared by sulfur-doped GO and octylamine has the smallest diameter of wear scar (0.25 mm) in the 928 lubrication oil at the concentration of 1 × 10^−4^ wt%, since the additive has a relatively high graphene weight percent (57.4 wt%), high sulfur content, (2.49 at%) and good dispersity (the octylamine has similar carbon chain length compared with the PAO4 or the base oil of 928 lubrication oil). Compared with the pure 928 lubrication oil and PAO4 oil, the decrement percent of wear scar diameter is 43.2% in the 928 lubrication oil and 17.2% in the PAO4 oil while the SA-GO modified by octylamine is applied with the concentrations of 2.5 × 10^−4^ wt% in PAO4 and 1 × 10^−4^ wt% in 928 oil, respectively. The tribological research of sulfur-doped graphene oxide suggests the SA-GO is an efficient anti-wear additive.

## Method

Sulfur-doped alkylated graphene oxide in this paper is prepared by the chemical modification of graphene oxide by P_4_S_10_ and four alkylamines (including octadecylamine, laurylamine, octylamine, and butylamine). Importantly, two different preparation routes are applied to obtain sulfur-doped alkylated graphene oxide by comparing mutually. One route that graphene oxide first reacts with P_4_S_10_ and then alkylamines results the product SA-GO, and another route that graphene oxide first reacts with alkylamines and then P_4_S_10_ gives the product AS-GO.

**Fig. 11:**
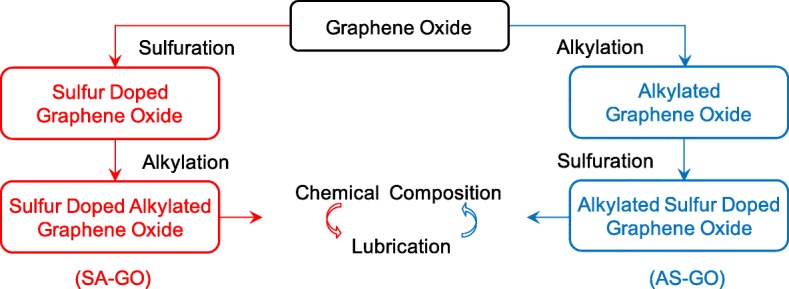
The contrastive research routes of SA-GO and AS-GO

As shown in Fig. 11, the research of SA-GO and AS-GO are designed to contrastively study the relationship between chemical composition and lubrication performance. The detailed experiment conditions are described as follows according to the preparation route of SA-GO.

### The Preparation of the Sulfur-Doped Graphene Oxide

In this paper, the sulfur-doped graphene oxide is prepared by the reactions between the P_4_S_10_ (chemical pure) and graphene oxide which is prepared by the modified Hummers’ method [33]. The detailed experiment condition is described as follow:

Three chemicals, 3 g graphite powder (3500 mesh, purity > 99.9 wt%, graphite used in the paper is commercially available in XFNANO Ltd. Co.), 1.5 g NaNO_3_, and 138 mL H_2_SO_4_, were added into a three-necked flask and stirred for 3 min. After being transferred to an ice bath, 3 g KMnO_4_ (chemical pure) was slowly added in the well-blended mixture to prevent the danger of overheating, then the mixture was placed in an oil bath to be refluxed and stirred for 6, 12, and 24 h, respectively.

The 100 mg as-prepared graphene oxide in the above procedure and 300 mg P_4_S_10_ were ultrasonically dispersed in 20 mL *N*,*N*-dimethylformamide (DMF) in a three-necked flask. Then the mixture was refluxed in N_2_ atmosphere at 100 °C for 24 h. After that, the temperature was allowed to cool down at room temperature and washed by acetone, alcohol, and DI water while negative-pressure filtration was carried out at the same time.

### The Preparation of the Sulfur-Doped Alkylated Graphene Oxide

The as-synthesized sulfur-doped graphene oxide (100 mg) was ultrasonically dispersed in 5 mL DMF and mixed with 20 mL SOCl_2_ (chemical pure) to react under a refluxing condition at 80 °C for 24 h. After being washed by tetrahydrofuran (THF) for removing SOCl_2_, the product ultrasonically dispersed in 2 mL THF which was added with 1 mL alkylamine (octadecylamine, laurylamine, octylamine, and butylamine, respectively), and the system was heated at 80 °C and stirred for 24 h. The light-yellow product, sulfur-doped alkylated graphene oxide, was thoroughly washed and vacuum-dried for further applications.

### The Characterization Instruments and Tribological Tests of the Sulfur-Doped Alkylated Graphene Oxide

Products in this paper were characterized by the attenuated total reflection Fourier transform infrared spectroscopy (ATR-FTIR, PerkinElmer Spectra Two), scanning electron microscopy (SEM, Hitachi SU-8000, secondary electron modes, acceleration voltage is 10 kV), transmission electron microscopy (TEM, TECNAI-F20 with accelerating voltage of 300 kV, bright field), selected area electron diffraction (SAED), Raman (Senterra&Veate X70, with excitation argon ion laser at 514.5 nm) and X-ray photoelectron spectroscopy (XPS, Escalab-250Xi; the curve fitting was done by using the Thermo Avantage v4.87 software based on Powell’s iteration method and 100 maximum iterations.), UV-vis spectrophotometer (Thermal Fisher, Genesys180), and TGA measurements were carried out on a TGA 8000 (PerkinElmer) analyzer from 50 to 550 °C under N_2_ with a heating rate of 10 °C/min.

The 928 aviation lubrication oil (commercially available in Henan Hangcai Science and Technology Co. Ltd.) and poly-α-olefin base oil (PAO, purchased from Shanghai Foxsyn Chemical Science and Technology Co. Ltd.) are applied as lubricants in tribological experiments. All of the sulfur-doped alkylated graphene oil samples were sonicated for 5 min before tribological tests. All of the tribological experiments were performed by a lever-type four-ball tester (Jinan Shijin Group Co. Ltd., MRS-10G and MRS-10P). The rotation speed of MRS-10G is 1450 r/min, the load is 392 N, and the testing time is 30 min; the rotation speed of MRS-10P is 1760 r/min and the testing time is 10 s. Steel balls used in the paper are uniform 12.7-mm GCr15 chrome steel ball which Rockwell hardness is 59-61HRC. The diameter of wear scar was measured by an optical microscope (resolution is ± 0.01 mm). All of the chemicals used in this paper are analytically pure except for the base oils, fully formulated lubricant oils, and the chemicals specifically stated.

## Supplementary information

**Additional file 1: Figure S1.** The relationship between the average friction coefficient and the concentration of AS-GO modified by butylamine, octylamine, laurylamine, and octadecylamine in PAO (a) and 928 (b) oils. **Figure S2.** The maximum nonseizure load (P_B_) value while SA-GO (prepared by sulfur-doped GO and butylamine (a), laurylamine (b), and octadecylamine (c), the oxidation time of the GO is 24 hours) is applied as lubrication additive with certain concentration in PAO4 base oil. **Figure S3.** The maximum nonseizure load (P_B_) value while SA-GO (prepared by sulfur doped GO and butylamine (a), laurylamine (b), and octadecylamine (c), the oxidation time of the GO is 24 hours) is applied as lubrication additive with certain concentration in 928 lubrication oil. **Figure S4.** The surface morphology and EDS spectrum of steel ball that pure 928 aviation lubrication oil is applied as lubricant

## Data Availability

The TG, TEM, and SEM data are available in the Analysis and Measurement Center of China University of Mining and Technology for inspection. The XPS, Raman, and line scanning EDX is available in the Analysis and Testing Center of Tianjin University of Technology for further inspection. Other data are acquired in Air Force Logistics College for inspection.

## Abbreviations

AS-GO: Sulfur-doped graphene oxide prepared by alkylation and then followed by sulfuration of graphene oxide
ATR-FTIR: Attenuated total reflection Fourier transform infrared spectroscopy
BN: Boron nitride
DMF: *N*,*N*-dimethylformamide
EDS: Energy dispersive X-ray spectroscopy
GO: Graphene oxide
GO-C12: The alkylated graphene oxide prepared by laurylamine and the sulfur-doped graphene oxide (the oxidation time of the graphene oxide is 24 h)
GO-C18: The alkylated graphene oxide prepared by octadecylamine and the sulfur-doped graphene oxide (the oxidation time of the graphene oxide is 24 h)
GO-C4: The alkylated graphene oxide prepared by butylamine and the sulfur-doped graphene oxide (the oxidation time of the graphene oxide is 24 h)
GO-C8: The alkylated graphene oxide prepared by octylamine and the sulfur-doped graphene oxide (the oxidation time of the graphene oxide is 24 h)
ILSAC: International Lubricant Standardization and Approval Committee
JCPDS: Joint Committee on Powder Diffraction Standards
PAO4: The poly-α-olefin base oil at 100 °C kinematic viscosity is ~ 4 mm^2^/s
SAED: Selected area electron diffraction
SA-GO: Sulfur-doped graphene oxide prepared by sulfuration and then followed alkylation of graphene oxide
SEM: Scanning electron microscopy
SiC: Silicon carbide
TEM: Transmission electron microscopy
TGA: Thermogravimetric analysis
THF: Tetrahydrofuran
UV-vis: Ultraviolet-visible spectroscopy
XPS: X-ray photoelectron spectroscopy
