# Supplementary material for: Sulfur-Doped Alkylated Graphene Oxide as High-Performance Lubricant Additive
Source: Nanoscale Res Lett. 2020 Jan 30;15:26. doi: 10.1186/s11671-020-3257-7 (PMC6990272; doi:10.1186/s11671-020-3257-7)
Supplement: Supplementary file 1 — Additional file 1: Figure S1. The relationship between the average friction coefficient and the concentration of AS-GO modified by butylamine, octylamine, laurylamine, and octadecylamine in PAO (a) and 928 (b) oils. Figure S2. The maximum nonseizure load (PB) value while SA-GO (prepared by sulfur-doped GO and butylamine (a), laurylamine (b), and octadecylamine (c), the oxidation time of the GO is 24 hours) is applied as lubrication additive with certain concentration in PAO4 base oil. Figure S3. The maximum nonseizure load (PB) value while SA-GO (prepared by sulfur doped GO and butylamine (a), laurylamine (b), and octadecylamine (c), the oxidation time of the GO is 24 hours) is applied as lubrication additive with certain concentration in 928 lubrication oil. Figure S4. The surface morphology and EDS spectrum of steel ball that pure 928 aviation lubrication oil is applied as lubricant [file 11671_2020_3257_MOESM1_ESM.docx]

**Additional file**

**^[[1]](#footnote-1)^**Sulfur Doped Alkylated Graphene Oxide As High Performance Lubricant Additive

Jun Ma^1,^*, Yunpeng Xiao^1^, Yuanbao Sun^1^, Jianqiang Hu^1^, Yuelun Wang^2,^*

^1^Air Force Logistics College, Xuzhou, 221000, People’s Republic of China;

^2^Key Laboratory of Coal Processing and Efficient Utilization, Ministry of Education, China University of Mining & Technology, Xuzhou, 221116, People’s Republic of China.

**1. The relationship between the average friction coefficient and the concentration of AS-GO in oils**

Figure S1 The relationship between the average friction coefficient and the concentration of AS-GO modified by butylamine, octylamine, laurylamine, and octadecylamine in PAO (a) and 928 (b) oils

**2. The maximum nonseizure load (PB) value of SA-GO in PAO4 and 928 oils**

Figure S2 The maximum nonseizure load (P_B_) value while SA-GO (prepared by sulfur doped GO and butylamine (a), laurylamine (b), and octadecylamine (c), the oxidation time of the GO is 24 hours) is applied as lubrication additive with certain concentration in PAO4 base oil

Figure S3 The maximum nonseizure load (P_B_) value while SA-GO (prepared by sulfur doped GO and butylamine (a), laurylamine (b), and octadecylamine (c), the oxidation time of the GO is 24 hours) is applied as lubrication additive with certain concentration in 928 lubrication oil

**3. The wear scar and EDS analysis of steel ball that pure 928 is applied**


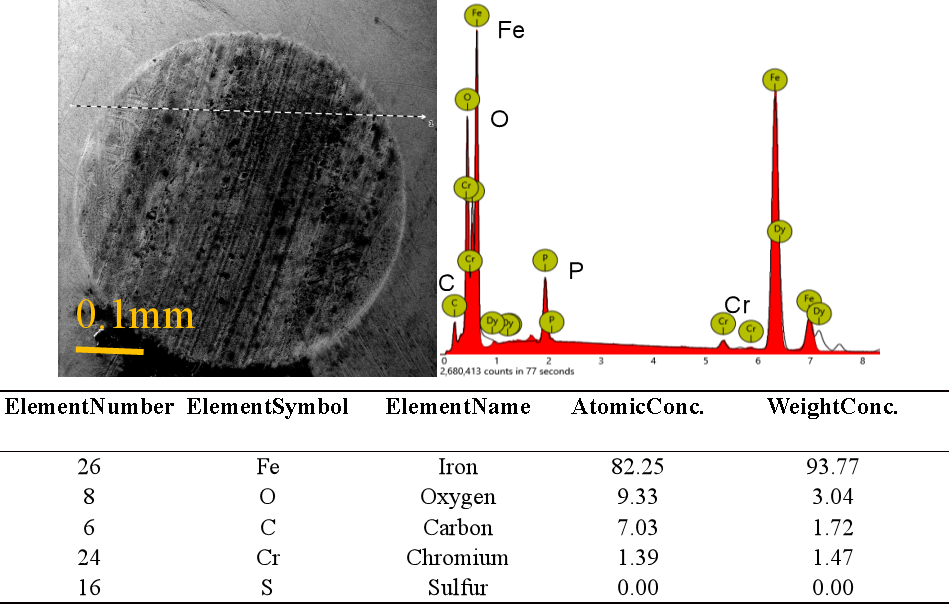


Figure S4 The surface morphology and EDS spectrum of steel ball that pure 928 aviation lubrication oil is applied as lubricant

1. *Corresponding author.

   E-mail: manudt@nudt.edu.cn (Jun Ma);

   E-mail: wangyuelun@126.com (Yuelun Wang). [↑](#footnote-ref-1)
